# Requirement of two simultaneous environmental signals for activation of Arabidopsis *ELIP2* promoter in response to high light, cold, and UV-B stresses

**DOI:** 10.1080/15592324.2024.2389496

**Published:** 2024-08-12

**Authors:** Okechukwu Samson Ezeh, Natsuki Hayami, Kana Mitai, Wasei Kodama, Satoshi Iuchi, Yoshiharu Y. Yamamoto

**Affiliations:** a The United Graduate School of Agricultural Science, Gifu University, Gifu, Japan; b Graduate School of Natural Science and Technology, Gifu University, Gifu, Japan; c Experimental Plant Division, RIKEN BioResource Research Center, Tsukuba, Ibaraki, Japan; d RIKEN CSRS, Suehiro-cho, Tsurumi-ku, Yokohama, Japan

**Keywords:** Synthetic promoter, transcriptional activation, stress signaling

## Abstract

Arabidopsis EARLY LIGH-INDUCIBLE PROTEIN 2 (ELIP2) is a chlorophyll- and carotenoid-binding protein and is involved in photoprotection under stress conditions. Because its expression is induced through high light, cold, or UV-B stressors, its mechanism of induction has been studied. It is known that a functional unit found in the promoter, which is composed of Element B and Element A, is required and sufficient for full activation by these stressors. In this study, the role of each element in the unit was analyzed by introducing weak mutations in each element as synthetic promoters in addition to intensive repeat constructs of each single element. The results suggest that a stressor like cold stress generates two parallel signals in plant cells, and they merge at the promoter region for the activation of *ELIP2* expression, which constitutes an “AND” gate and has a potential to realize strong response with high specificity by an environmental trigger.

## Introduction

For plants, photosynthesis is essential but can also be detrimental because it has the potential to induce reactive oxygen species (ROS) in plant cells under undesirable conditions, such as high intensity of sun light (HL), cold temperature, high temperature, drought, submergence, and nutritional deficiency. Facing these risks, plants have developed anti-stress factors that suppress the damage. Because these factors also suppress photosynthesis and plant growth, their expression is often tightly regulated. Their regulation is not only a matter of survival but is also a key issue for plant growth under changing environmental conditions.

EARLY LIGHT-INDUCIBLE PROTEIN (ELIP) is a chlorophyll *a*/*b*-binding (CAB) super family protein that binds chlorophylls and carotenoids.^1^ While the light-harvesting group of the superfamily promotes photosynthesis, another group that includes ELIP, PsbS, LiL, SEP, and OHP/HLIP plays a role in photoprotection.^2,3^ Although their exact molecular functions are yet to be elucidated, PsbS has been found to have a role in the dissipation of excess energy from Photosystem II.^4^

Consistent with its suggested role, ELIP genes are activated by environmental stressors, including HL in peas^5^ and barley^6^; UV-B in peas^7^ and desiccation in a resurrection plant^8^; and cold temperature in peas.^9^ In the case of the evergreen tree *Rhododendron catawbiense*, the development of frost tolerance was found to have a positive correlation with the *ELIP* expression levels in a field study.^10^

In addition to gene expression, the number of ELIP genes in a genome was reported to have a correlation with stress tolerance in some studies. A recent comparative genomics study of two close grass species, desiccation-sensitive *Sporobolus pyranudakus* and desiccation-tolerant *S. stapfianus*, revealed a drastic increase in *ELIP* copy number in the tolerant species.^11^ Together with the strong induction profiles for stress perception, ELIP is suggested to have a critical role in stress tolerance, potentially through photoinhibition relief as a pigment-binding thylakoid protein.

Arabidopsis ELIP expression is also induced by environmental stressors that cause photoinhibition. Its expression is activated through HL,^12^ UV-B,^13^ and cold temperature stressors.^14^ To understand the mechanism of gene expression activation by the multiple environmental stressors, the Arabidopsis *ELIP2* promoter was analyzed with the aid of a promoter prediction methodology we previously reported.^14^ Experimental validation using native and synthetic promoters revealed that a functional unit composed of two distinct elements in the promoter, Element B and Element A, were necessary and sufficient for the multiple stress responses of *ELIP2*, and that the functional unit was composed of two *cis*-elements.^14^

The combined function of the two distinct *cis*-regulatory elements is also reported in the ABA response of the barley *HVA22* promoter, which revealed the Coupling Element (CE) to support the ABA-Responsive Element (ABRE).^15^ Another example was found in a synthetic promoter study using transgenic Arabidopsis to evaluate several known light-responsive elements, which revealed multiple combinations of two *cis*-elements to reconstitute light responsiveness and green tissue-specific expression.^16^ The importance of the combined function of the two *cis*-elements is also highlighted in the prediction of stress responsiveness in Arabidopsis based on the promoter sequences.^17^ While these reports have demonstrated the importance and generality of the combined function of the two *cis*-elements, their molecular mechanisms remain unknown.

Regarding the role of Element B found in the *ELIP2* promoter, previous *in vitro* and mutant analyses revealed that it is recognized by HY5,^14^ which is activated by blue light signals through CRY1^18^ and UV-B signals through UVR8.^19^ While UVR8 mediates a signal that is considered as a stress signal, HY5 does a typical light signal that is initiated from phytochromes and cryptochromes,^20^ which is activated by weak light. Therefore, the finding of HY5 in mediating the light stress activation of *ELIP2* raised a new conundrum about what the difference in the signal transduction between a light signal promoting photosynthesis and a light “stress” signal repressing it is. In addition, it is suggested that Element B receives cold stress signals based on the promoters having it; however, to date, there is no direct evidence of Element B receiving the cold signals.

In this study, we considered the mechanism of stress activation of *ELIP2*, which is suggested to induce photoprotection of photosynthetic apparatus. Here, we address the framework of the promoter structure, which enables stressor-specific activation through the modulation of the functional unit in the promoter.

## Materials and methods

### Synthetic promoter construction

For the preparation of the synthetic promoters, Element A [5’-acTACACACCac-3’], Element B [5’-gaGGCCACGCCAtc-3’], and their respective point mutations were triplicated with a spacer [5’-AAAA-3’] between them. These were inserted into the upstream region of the 35S minimal promoter-luciferase fusion of yy447^14^ (Figure 2). Detailed information regarding the vector is available on the website (https://www1.gifu-u.ac.jp/~yyy/yyy/plasmid.html). Synthetic promoters containing only Element A or Element B were prepared as described.^14^

### Plant transformation and establishment of transgenic lines

Binary vectors harboring the constructs were introduced into *Arabidopsis thaliana* (Col-0) using *Agrobacterium*-mediated transformation, and two to four independent T_2_ lines with resistance to phosphinothricin (10 ug mL^−1^) were subjected to analysis as previously described.^14^ Point mutation series (yy664 to 672) have single T-DNA insertion for each line, checked with competitive PCR.^14,21^ Copy number of transgenic plants for 6×A and 6×B constructs was not examined.

### Stress treatments

Arabidopsis seeds were surface-sterilized and stratified at 4°C for 2–3 days in the dark before seeding them on germination medium. Thirteen seedlings per line were grown in a 30-mm diameter culture dish containing 10 mL of germination medium supplemented with 1% sucrose for 8 days under continuous white light (6 W m^−2^ = 30 uE m^−2^ s^−1^) at 22°C.

Eight-day-old seedlings were exposed to the respective stressor, and the bioluminescence was measured. HL irradiation for 3 h at 150 W m^−2^ was achieved using a 1,000-W xenon lamp as previously described.^22^ UV-B treatment was achieved using fluorescence tubes (Ultraviolet B TL20W/12RS, Phillips Electronics, Netherlands) for 12 min at a light intensity of 6 W m^−2^ measured using a Light Meter (LI-250A, LI-COR Bioscience, USA). After UV-B treatment, the seedlings were returned to the continuous white-light condition. HL and UV-B treatments were performed at 22°C. Cold treatment was performed in a growth chamber (MIR-154-PJ, Panasonic Healthcare, Japan) for 24 h at 4°C under continuous white light at a light intensity of 6 W m^−2 14^. Individual lines per construct were assayed in triplicate.

### In vivo luciferase assay

Eight-day-old seedlings in 3 cm-dishes containing GM media supplemented with 1% sucrose and 0.8% agar were sprayed with 1% (w/v) luciferin (Dojindo, Kumamoto, Japan) and 0.01% (v/v) Triton X-100 one day prior to starting the various treatments^22^ and then assayed with a photomultiplier-based automated luminescence counter (LL04–1, Churitsu Electric, Nagoya, Japan).^14^ The counter measures bioluminescence in a dark chamber for 1 min after dark adaptation for 1 min to release delayed fluorescence from chlorophylls. Except for the time in the dark chamber, dishes were illuminated with continuous white light at a light intensity of 6 W m^−2^. The measurement was automatically repeated with 10 ~ 30 min intervals. The numbers of utilized transgenic lines for each construct are: 1, 4, 4, 2, 2, 4, 4, 4, and 4 for yy468, 664, 666, 667, 668, 669, 670, 671, and 672, respectively.

Luminescence images were obtained one day after luciferin feeding with an EM-CCD camera (C9100–13, Hamamatsu Photonics, Hamamatsu, Japan) set in a dark chamber as described previously.^23^

### Real-time qPCR

Total RNA was extracted from whole plants of control and stress-treated plants of the wild type (B+A) and other synthetic promoter constructs and subjected to real-time quantitative – PCR analysis as described previously.^24^ Primers used for RT-qPCR are LUC_F (5′-TGGCTATGGCTGAAGACGCC-3′), LUC_R (5′-GCTCTCCAGCGGTTCCATCT-3′), UBQ5_F (5′-ACAAGAAAAACAAACCCTATCAAAGG-3′), and UBQ5_R (5′-AGAGCTGTCAACTGCAGGAAGAA-3′).

## Results

### Functional unit of two elements

**Figure 1.**
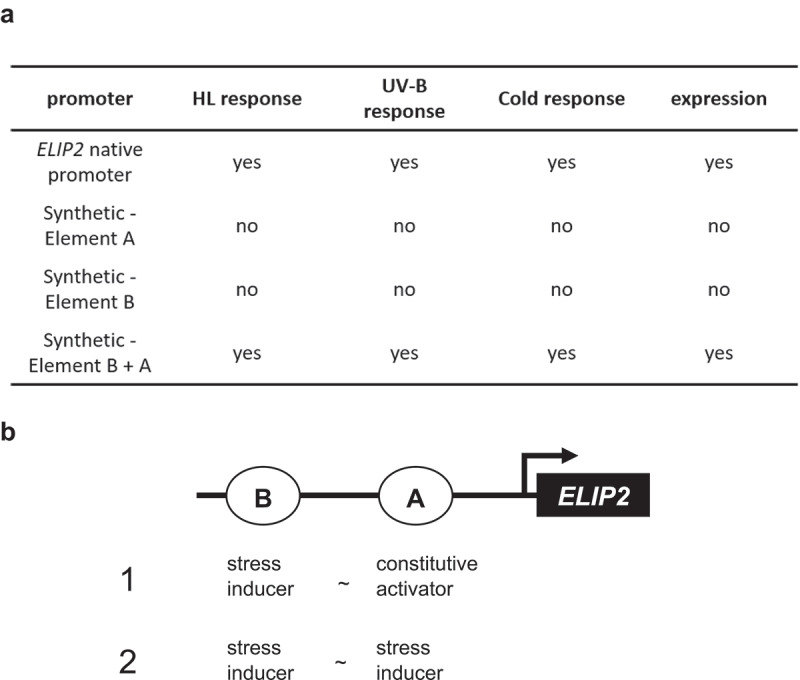
Functional unit of element B and Element a mediating the HL, UV-B, and cold stress responses of *ELIP2* expression.

Figure 1(a) summarizes the development of synthetic promoters according to previous studies.^14^ The 1 kb-long native promoter represents the expression profile of *ELIP2* and was activated by HL, UV-B, and cold stressors. This expression profile was represented by a synthetic promoter composed of Element B and Element A but ones that were composed of only one element did not show sufficient promoter activity to exhibit a stress response.

(a) Summary of the promoter analysis reconstructing the stress response of the native *ELIP2* promoter^14^. (b) Possible roles of the two elements in stress activation. Both Element B and Element A are required for transcriptional activation, and thus their possible roles here should be either as a stress inducer or as a constitutive activator. Element B accepts environmental signals through HY5 ^14^; therefore, it is a stress inducer. Regarding Element A, no suggestive data is known, except for the observation that it is found in promoters less specific to the stress responses in Arabidopsis genome than Element B. The molecular function of transcriptional activation is not considered here, and the models are provided to explain the signal mediation to the promoter.

As mentioned in the Introduction, Element B is recognized by HY5, which is a signal mediator from CRY1 and UVR8. Therefore, Element B was a target site for the environmental signals. Considering this, there are two models regarding the role of the two elements, which is shown in Figure 1(b).

One model assumes that Element A is a constitutive activator that supports Element B, and without this element, the promoter cannot be sufficiently activated at detectable levels (1 in Figure 1(b)). Another model hypothesizes that Element A is a stress inducer (2 in Figure 1(b)). In this model, the promoter receives two parallel stress signals for its activation, and the stimulation of a single element is not sufficient for the detection of transcriptional activation. In this report, we address these possibilities through the functional analysis of synthetic promoters.

### Partial suppression of each element in the functional unit

**Figure 2.**
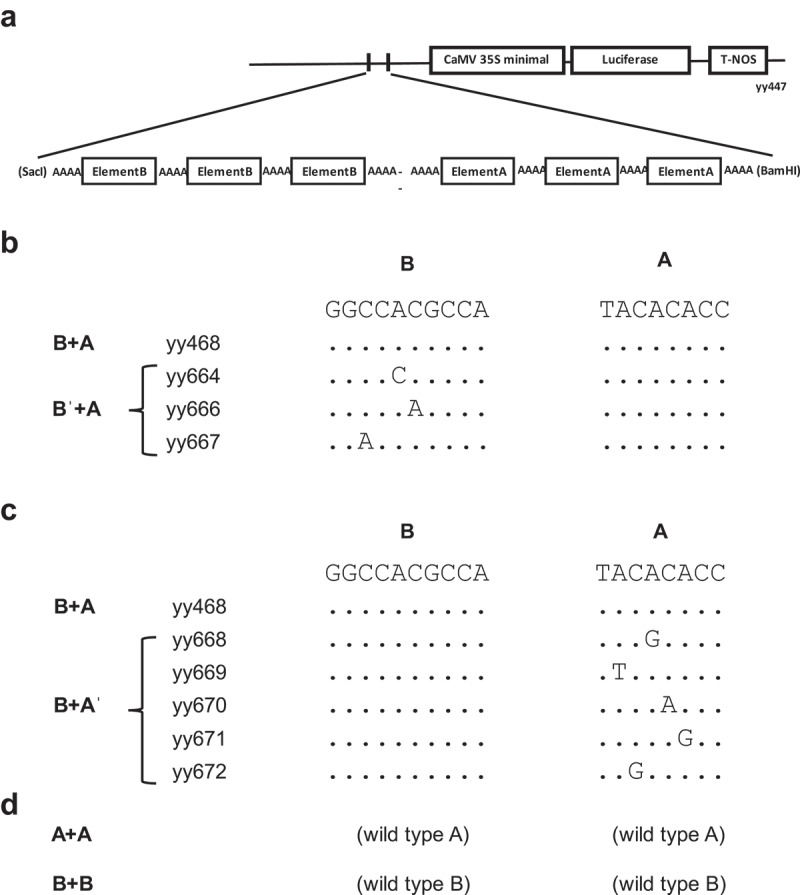
Constructs and sequences of synthetic promoters for the mutation series of element B.

Figure 2(a) shows “wild-type” synthetic promoter that is composed of Element B and Element A. Downstream of the unit is a CaMV 35S minimal promoter (−46) that contains a TATA box and a transcription start site, coding region of a luciferase reporter, and NOS terminator.^14^

(A) Reporter constructs used in this study. Synthetic oligonucleotides containing the two elements were inserted into the *SacI/BamHI* site of the luciferase reporter gene. CaMV 35S minimal is “-46” including a TATA box and Inr. T-NOS is a NOS terminator sequence that includes the polyA site. (b) Sequences of the mutated Element B used in the study. The design of the point mutations was determined to provide a weak reduction in Element B function based on the results of promoter prediction. The three repeats of the element in the construct are the same sequence as shown in the panel. All the mutated constructs are accompanied by the non-mutated (wild-type) Element A. (c) Sequences of mutated Element A. (d) Single element constructs. They have 6× Element A (3 × A + 3 × A) or 6 × Element B (3 × B + 3 × B), both of which dissolve the combination.

Functional evaluation of each element is normally performed by disruption of one of the elements in the combination. However, complete disruption causes the loss of promoter activity (Figure 1(a)). In this study, we introduced point mutations in the combination constructs and expected partial suppression of the function of each element.

Figure 2(b) illustrates the mutations in Element B in the unit prepared for this study. We introduced weak mutations by consulting the signals of promoter prediction data.^25^ All the Element B mutations were paired with the wild-type Element A. Figure 2(ac) illustrates the mutations in Element A.

In addition to the series of point mutations, element substitution constructs, “A + A” and “B + B” were prepared instead of 3 × A or 3 × B, respectively; therefore, “B +A,” “A + A,” and “B + B” means “3 × B + 3 × A,” “3 × A + 3 × A = 6 × A,” and “3 × B + 3 × B = 6 × B,” respectively.

These reporter constructs were introduced into Arabidopsis v*ia* Agrobacterium to establish the stable transgenic lines.

### Element B partial suppression caused a reduction in stress responses

**Figure 3.**
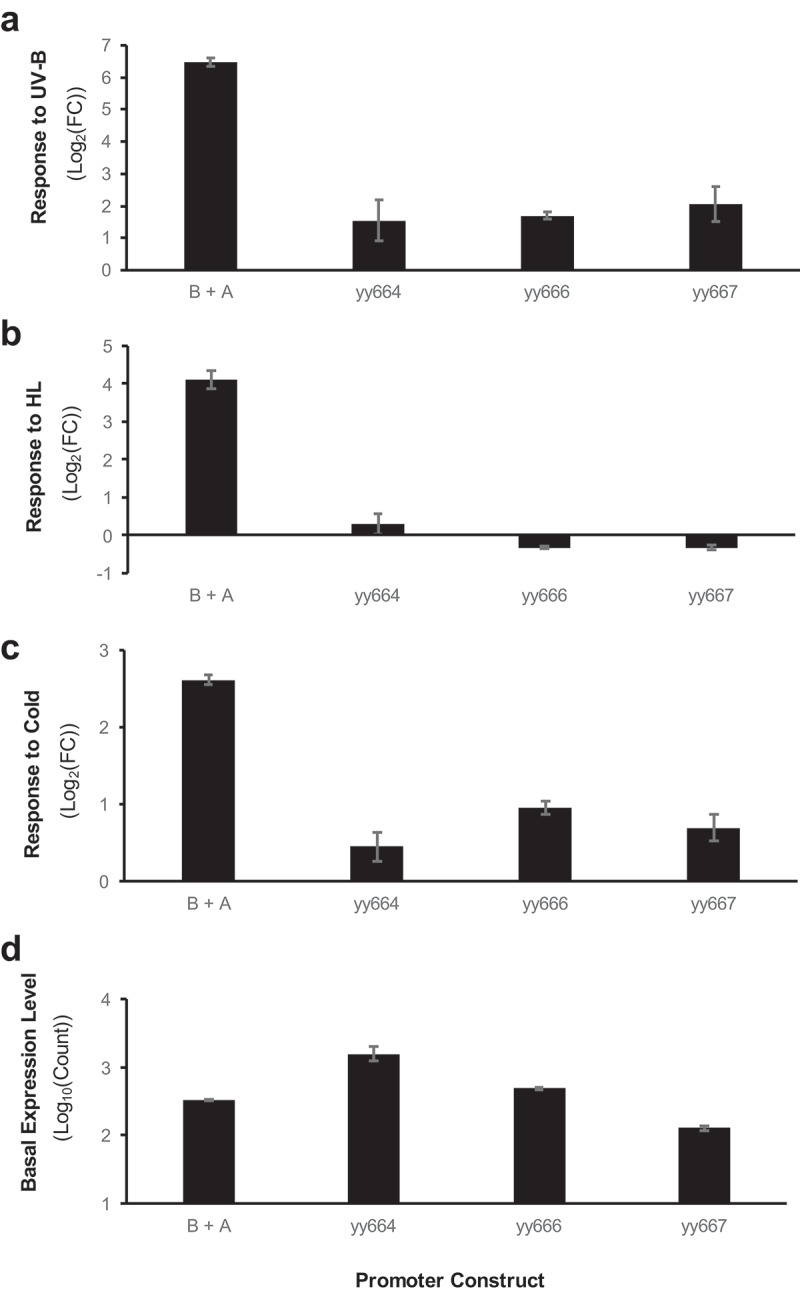
Effect of point mutations in element B under the B + a synthetic promoter conditions.

We first analyzed the effect of the point mutations in Element B in the unit (B’ + A, Figure 2(b)). Figure 3(a) shows its response to UV-B stimulation. The vertical axis of the graph indicates a log_2_FoldChange (FC). Because of the partial suppression of Element B, we were able to detect reporter activity before and after the stress treatment to measure the stress response. The high response to UV-B stimulation in the wild-type unit (B + A) was reduced by mutations in Element B (yy664-yy667), as shown in the panel. Similarly, the mutation series revealed a reduction in stress response to HL stimulation (Panel B) compared with the wild-type unit. These results indicate that Element B receives stress-dependent activation signals. The acceptance of the HL and UV-B signals by Element B is consistent with the role of the element as an HY5 recognition site, which mediates environmental signals from CRY1 and UVR8.^14^

(a) Response to a UV-B pulse (6.5 W m^−2^ for 12’) under weak white light conditions. (b) Response to HL (150 W m^−2^ for 3 h). (c) Response to cold treatment (4°C for 24 h) under weak white light conditions. In each experiment, eight-day-old seedlings were exposed to the corresponding treatment condition. Data are presented as the mean and standard error (SE). The introduced mutations for each construct are shown in Figure 2.

Panel C shows the cold responses of the mutants. As shown, mutations in Element B also resulted in reduction of the cold stress response. These results revealed that Element B receives the cold signal as well, which is a new finding of this report.

### Element A partial suppression caused a reduction in stress response

**Figure 4.**
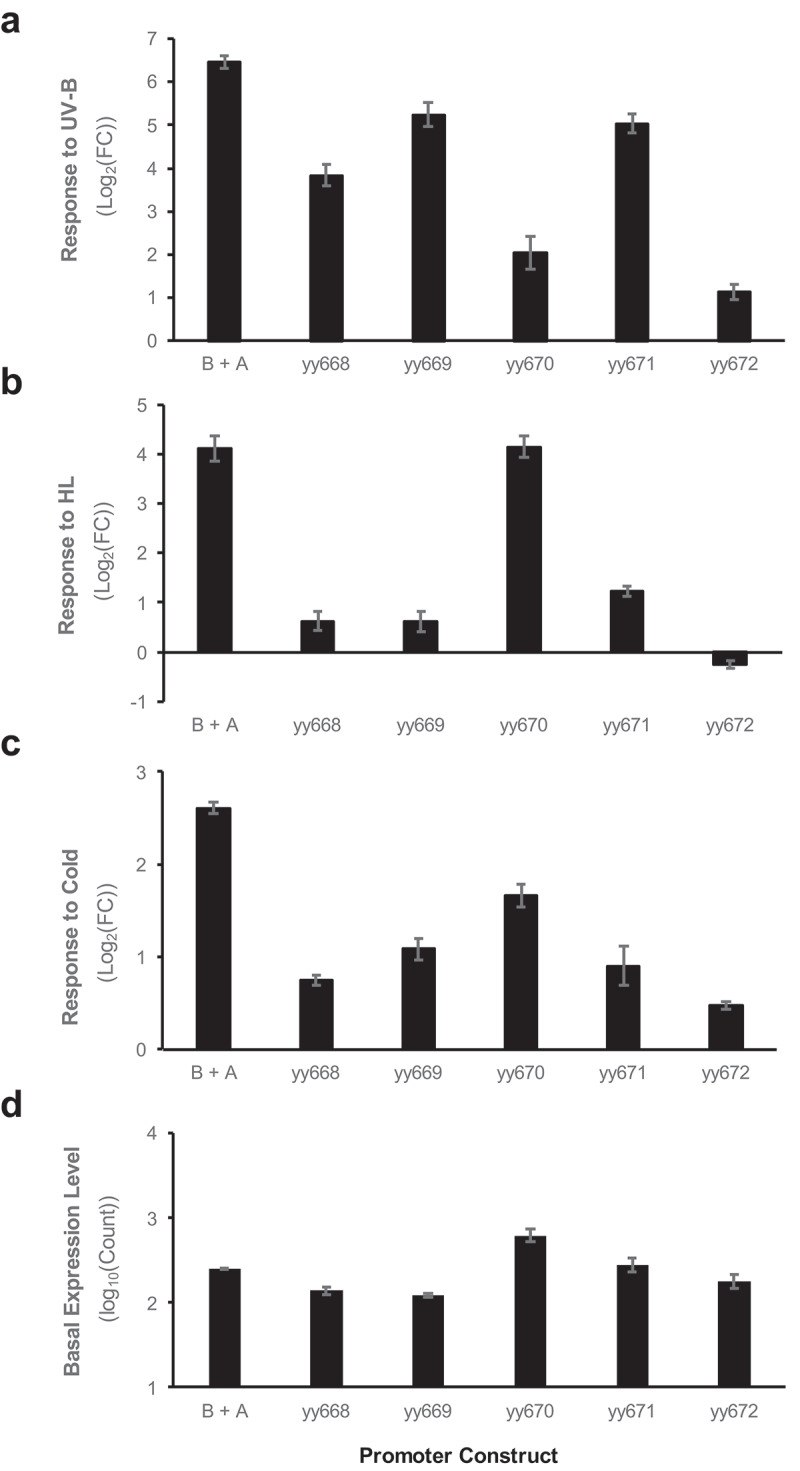
Effect of point mutations in element a under the B + a synthetic promoter conditions.

Change in the stress response by the mutation of Element A is shown in Figure 4. The five mutant constructs that were analyzed exhibited different profiles; however, they all reduced by at least a FC compared with the wild-type (B + A) in some or all of the stress responses. The observed reduction revealed that Element A also receives stress signals, in addition to Element B. Therefore, these results support the second hypothesis in Figure 1(b).

Response to a UV-B pulse (6.5 W m^−2^ for 12’) under weak white light conditions. (b) Response to HL (150 W m^−2^ for 3 h) conditions. (c) Response to cold treatment (4°C for 24 h) under weak white light conditions. In each experiment, eight-day-old seedlings were exposed to the corresponding treatment condition. Data are presented as the mean and standard error (SE). The introduced mutations for each construct are shown in Figure 2.

**Figure 5.**
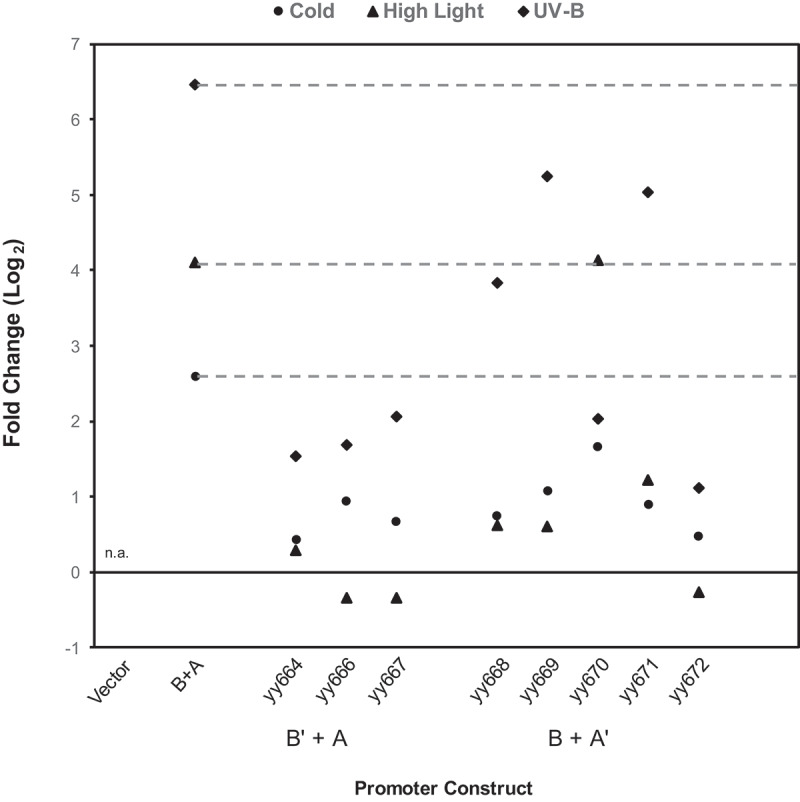
Summarized results of the effect of point mutations in element B and Element a under each synthetic promoter condition.

The summarized results of the stress responses of B’ + A and B + A’ are shown in Figure 5. As shown in the graph, the B’ + A series reduced the FC simultaneously under all three stress responses, regardless of the mutations. This result indicates that uniform recognition mediates all three stress signals. If HY5 alone is the corresponding transcription factor, these results are reasonable. If an addition transcription factor(s) is involved in the recognition of Element B, these results suggest that the transcription factor(s) and HY5 have similar target sequence preferences.

Dotted lines show the level of responses of the wild-type condition (B + A) for the cold, HL, and UV-B stressors, respectively. The introduced mutation for each construct is shown in Figure 2

In contrast, the responses of B + A’ did not exhibit uniform reduction but varied according to the constructs. The constructs yy669 and yy671 showed a harsh reduction in their responses to cold and HL, but the reduction of the constructs to UV-B was moderate. However, yy670 exhibited a different profile, with reduced responses to cold and UV-B but maintaining a conserved response level to HL. It was also noted that all five mutants showed a substantial reduction in their response to cold stimulation. These analyses revealed that the different mutations in Element A resulted in a spectrum of different stress responses. This suggests that Element A is recognized by multiple transcription factors with different sequence preferences, which mediate different stress signals.

### Functionality of single elements

The functional unit in our synthetic promoters, B + A, consists of a triplicate Element B and a triplicate Element A. Element B was then substituted for Element A to create 6× Element A repeats to generate the element (A + A), as shown in Figure 2(d). The other substitution of the unit was prepared to make a 6× repeat of Element B to generate the element (B + B). In general, the increase in the copy number of transcriptional regulatory elements results in an increase in the expression level.^26^ Therefore, these substitutions have the potential to elevate the expression of the reporters to detectable levels.

The UV-B response of A + A was investigated. As shown in Figure 6(a), A + A showed a positive response to UV-B stimulation, while “A” with only 3× repeats failed to exhibit a response because of low signal levels. The observed positive response by A + A is consistent with the finding that Element A also receives stress signals, as shown in Figure 5. Similar results were obtained with Element B, where B + B showed a high response to UV-B without the presence of Element A (Figure 6(b)).

**Figure 6a.**
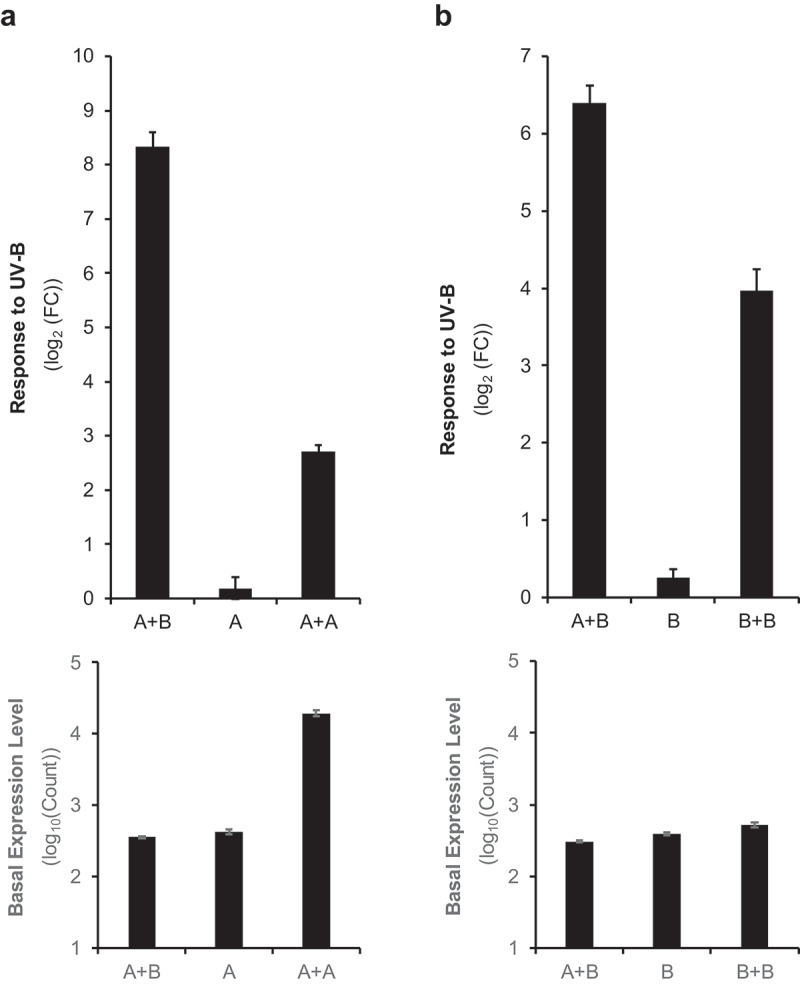
Partial activation by UV-B in the “A + A” and “B + B” constructs.

Response at 3 h after the UV-B pulse under the white light conditions is shown as the average (log_2_FoldChange) and standard error. See Figure 2 for constructs. C – E Imaging analysis of reporter activity driven by synthetic promoters. Positions of the constructs shown in Panel C are kept in Panels D and E as well. Image of (C) seedlings taken with a digital camera. Luminescence images of seedlings with pseudo color before (D) and after (E) UV-B stress.

**Figure 6b.**
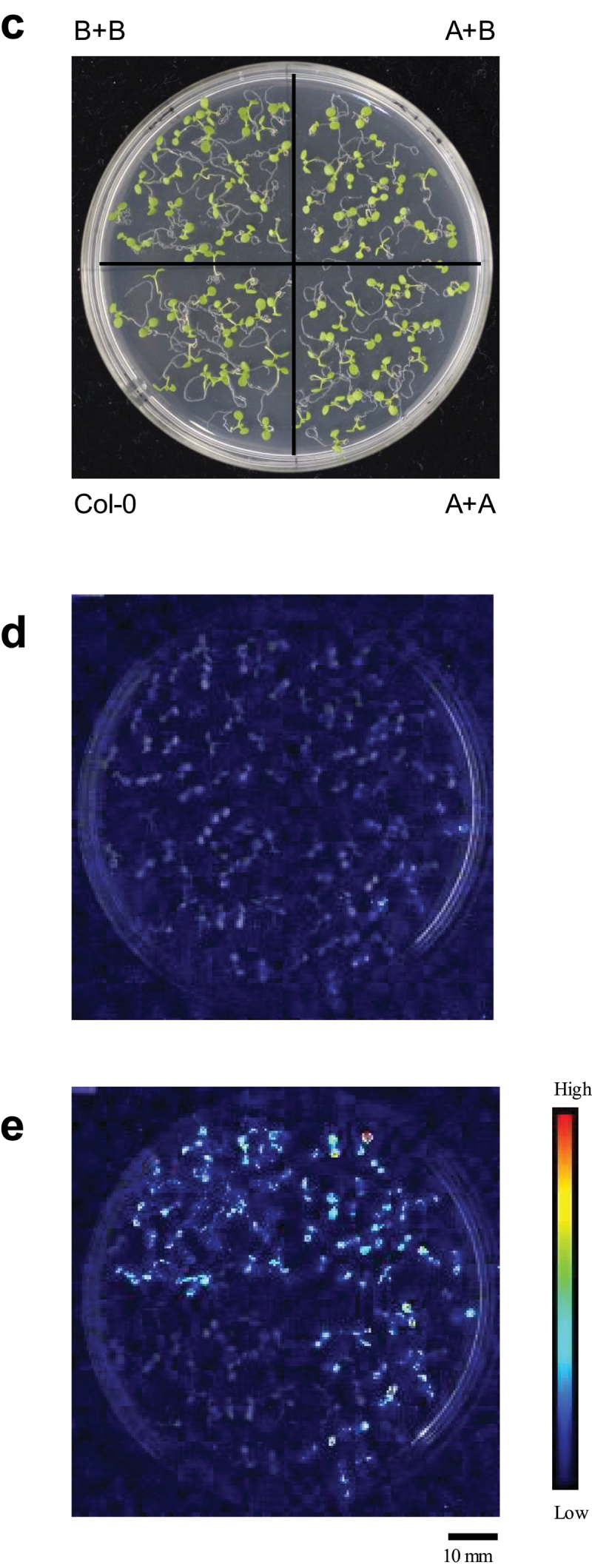
(Continued)

The responsiveness of A + A and B + B can be observed visually with high-performance CCD camera. As shown in Figure 6(d) (before stress) and 6E (after stress), UV-B treatment caused stronger luminescence signals in the whole cotyledon for both A + A and B + B, consistent with the results obtained by the photon counting method (Figure 6(a,b)).

Consistent with the results of the photon counting method and imaging analysis of *in vivo* luciferase assay as described above, transcript analysis of the reporter gene by RT-qPCR confirmed UV-B activation of the A + A and B + B constructs (Fig S1).

These results revealed the autonomous functionality of Element A and Element B. These elements were able to function alone to activate transcription if there was a sufficient number of copies in a promoter, which was more than three. At the same time, the results confirmed the input of the stress signals to Element B and Element A, thus providing an additional line of evidence.

It was also noted that the degree of responses of A + A and B + B were considerably lower than that of B + A, thereby demonstrating the synergistic action between Element B and Element A. This synergy of the unit contributes to the high response to environmental signals, which was not possible with only a single element.

### Element B and G-box

The HY5-bindng motif, G-box, is known as an element that promotes photomorphogenesis and is found in promoters of light-harvesting protein genes, genes for the small subunit of RuBisCO, and other light responsive genes that promote photosynthesis.^27,28^ Our previous study revealed that HY5 binds to Element B,^14^ which cannot be categorize into G-box because of the sequence. To understand the difference between Element B and G-box, the distribution of Element B and G-box among the Arabidopsis promoters in the genome was compared.

Table 1 shows the stress responses of the promoters that contain G-box, Element B, and a consensus sequence of a HY5 target determined by *in vitro* binding assays. There are several definitions of G-box, and in this study we selected an octamer [5’-ACACGTGG-3’] from a longer definition [5’-TGACACGTGGCA-3’] to include the core ACGT (underlined). Compared with Element B and G-box, the HY5 target defined by Kurihara et al. [5’-(G/T)(C/A)CACGT(C/G)-3’]^29^ shown in the table is the least stringent definition of the HY5 targets.

**Table 1.** Average of stress response of genes containing the HY5 target, G-box, and element B in the promoter region.

	HL	Cold	UV-B
All (12,975)	−0.0978	−0.0651	−0.0676
HY5 target (2,446)	0.00956	0.0516	−0.0795
G-box (644)	0.0648	0.147	−0.0970
Element B (28)	0.468	0.277	0.443

A figure in parentheses indicates the number of promoters that contain the corresponding element. Values indicate log_2_ (FoldChange). The motifs used in the table are as follows. HY5 target: [5’-(G/T)(C/A)CACGT(C/G)-3’].^29^ G-box: [5’-ACACGTGG-3’] (core octamer was selected from Chattopadhyay et al.^27^ The direction of the element sequence was not considered in all cases.

The stringency of the motifs is reflected by the number of genes that contain the motifs within their promoter region (−1,000 to −1 relative to the transcription start site), where a larger number represents a less stringent motif. Element B is not included in either G-box or the HY5 target because it has a critical mismatch within the core region of these motifs and the conserved region of the bZIP family in higher plants,^30^ which is ACGT.

Table 1 shows the average of the log_2_FC of the promoters that contain the HY5-related motifs. All the Arabidopsis genes that were expressed in the HL,^31^ cold,^32^ and UV-B^32^ expression analysis, and whose transcription start site data are available,^33^ were subjected to the analyses. The average response to HL, cold, and UV-B stimulation were −0.0978, −0.0651, and −0.0676, respectively. Compared to these, the HY5 target group had slightly higher responses to HL and cold but had no increase in response to UV-B stimulation. The G-box group had a higher response to HL and cold than HY5 target, and had no increase in response to UV-B stimulation. Compared with these motifs, the promoters that contained Element B had substantially higher responses to these stressors. As shown in Table 1, the responses of the group of Element B to HL, cold, and UV-B were 0.468, 0.277, and 0.443, which are highest among the four groups.

Table 2 shows the degree of the response to the stressors through the rates of the stress-responsive genes within the four groups. The tendency of the HL and cold responses was the same as the average of the responses (Table 1), which is a slight increase in the HY5 target and G-box and a substantial increase in Element B. The trends of the UV-B responses were not exactly the same as the average comparison, but Element B still had the highest ratio of the stress-responsive genes.

**Table 2.** Ratio of the stress-responsive genes among the gene groups containing the HY5 target, G-box, and element B in their promoter region.

	HL	Cold	UV-B
All	3.21%	6.49%	8.74%
HY5 target	5.76%	10.3%	9.12%
G-box	6.99%	12.4%	10.2%
Element B	21.4%	21.4%	21.4%

Genes showing a positive response (FoldChange >2.0) were considered stress-responsive in the table. See Table 1 for details.

In our previous study,^14^ Element B was found in the promoters that had stress responses but also in those that exhibited no stress responses or negative responses. Among them, the promoters that displayed a positive response to HL also showed a positive response to cold and UV-B stimulation, and those that showed a negative response to HL also had a negative response to cold and UV-B stimulation. The analysis revealed a high correlation between HL, cold, and UV-B responses among the promoters that contained Element B.

Table 3 shows the correlation analysis between these stress responses among all the Arabidopsis promoters that are expressed and contains the HY5 target, G-box, and Element B. Consistent with our previous analysis, promoters that contain Element B show a high correlation between HL and cold, HL and UV-B, and cold and UV-B. However, promoters that contain G-box or the HY5 target demonstrated a lower correlation. This analysis also revealed more involvement of Element B in the stress responses than that of G-box or the HY5 target.

**Table 3.** Correlation between two stress responses containing the HY5 target, G-box, and element B in their promoter region.

	HL ~ Cold	HL ~ UV-B	Cold ~ UV-B
All	0.199	0.192	0.307
HY5 target	0.254	0.218	0.272
G-box	0.246	0.224	0.204
Element B	0.865	0.786	0.737

The four groups of genes were subjected to correlation analysis between HL and Cold responses, HL and UV-B responses, and Cold and UV-B responses. The correlation coefficients are shown in the table. See Table 1 for details.

In summary, the results shown in Tables 1–3 suggest that the genes containing Element B in the promoter region are considerably enriched in HL, cold, and UV-B stress responses, which was not observed in the HY5 target and G-box groups. Element B connected these three stress responses, which were not observed in the cases of the HY5 target and G-box. The highest stress response of the Element B group cannot be explained only by the involvement of HY5 for its function but suggests the involvement of other factors that are related to the stress response.

## Discussion

**Figure 7.**
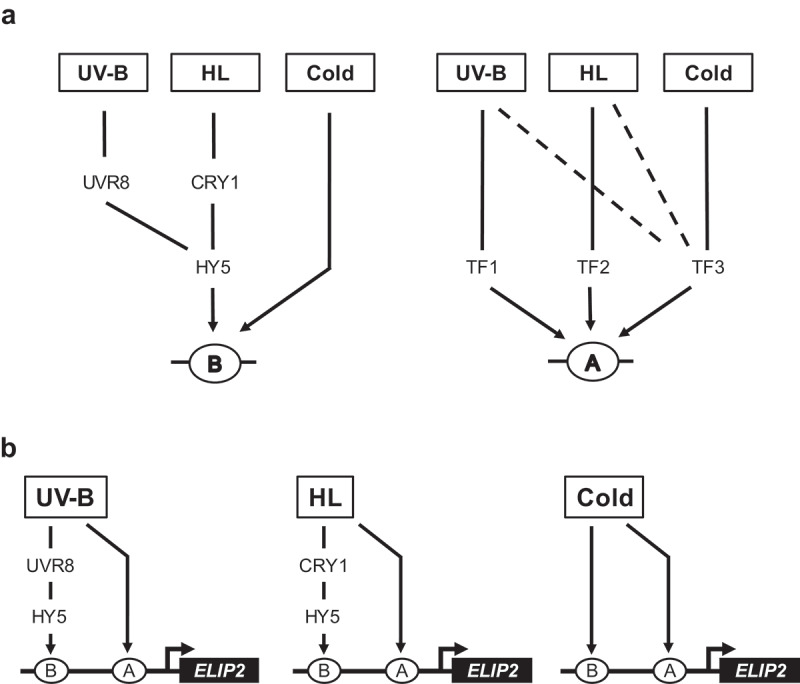
Signal mediation for the ELIP2 promoter.

The stress responses to HL, cold, and UV-B stimulation through the expression of *ELIP2* are operated by a functional unit composed of Element A and Element B. In this study, we revealed their individual roles in stress responses by the synthetic promoter approach. They can function individually if they are repeated many times in the promoter, but with a limited number of repeats, both elements alone are not enough to activate the promoter, such as the *ELIP2* promoter. Stress signaling to the *ELIP2* promoter is illustrated in the two panels of Figure 7 with emphasis on the different aspects.

(A) Multiple signals converge at Element B and Element A. Element B receives signals that are mediated by HY5. The specific distribution of Element B for HL, cold, and UV-B responsive promoters suggests the involvement of the element in receiving the cold signal. Mutation of HY5 does not cause a considerable reduction in the cold response 14, which suggests the involvement of another transcription factor(s) that is independent of HY5 for the cold response of the element. However, its target sequence preference is suggested to be similar. Element A is suggested to be bound by at least three transcription factors (TFs). In addition to the wild-type Element A, yy669 and yy671 were preferentially bound by TF1, while yy670 was preferentially bound by TF2. (B) Two parallel environmental signals are required to mediate the promoter for each response.

## Single regulatory element is recognized by multiple transcription factors for multi-stress response

Figure 7A focuses on the flow of input signals to each element. Element B is an HY5 recognition site for signal transduction from CRY1 for HL and from UVR8 for UV-B stimulation.^14^ Mutant analyses revealed that the cold signal was not mediated by HY5 but rather by an unidentified transcription factor. These results revealed that one of the unidentified factors binds to Element B with a similar sequence specificity to HY5.

The stress response under the control of a light signaling factor, HY5, remains to be fully elucidated. The light signal for the CRY1-mediated inhibition of hypocotyl elongation is saturated at ~ 1 uEm^−2^s^−1^,^34^ while the light stress signal for *ELIP2* activation is at ~ 750 uEm^−2^s^−1^ (150 W m^−2,22^ thus revealing a ~ 3 order difference in their sensitivities. In addition, a large number of photosynthesis-related genes, including *PSAD*, *PSAF*, *PSBT*, and many light-harvesting complex (LHC) genes, which are known to be activated by light signals, are suppressed under HL.^23^ Therefore, the light response in photomorphogenesis and the light stress response should be physiologically different.

Because Arabidopsis *hy5* mutants were first isolated with the phenotype of defective photomorphogenesis,^35^ major targets of HY5 had been considered as photomorphogenesis-promoting genes as mentioned. However, additional target genes have been identified, including genes for the nutrient transport and sugar metabolism.^36^ In the case of regulation of a nitrate transporter gene, *NRT2.1*, HY5 is supposed to mediate a light-activated signal related to C/N balance.^37^ These recent findings extended the role of HY5 from promotion of photomorphogenesis to a broader one which includes modulation of gene expression under changing light and nutritient conditions. Currently it is not known if the C/N balance or nutritional deficiency affects HL stress response.

One possible mechanism for the light stress-specific response that is dependent on Element B is its combined function with Element A to modulate the response specificity. In addition, there is the possibility that Element B itself has a stress-specific function that is not shared with G-box. The latter possibility is supported by the substantial enrichment of the stress responsive genes in the Element B-containing group (Tables 1 and 2) and the unification of the three responses in the Element B-containing group (Table 3). These characteristics of the promoter group are evidently different from those of the HY5 target or G-box (Tables 1–3).

These differences can be explained by involvement of an additional transcription factor(s). In the case of G-box, it is also known to be the binding site of HYH and GBFs.^38^ Therefore, G-box is not just the target of HY5 but has additional roles in receiving environmental signals. HY5, HYH, and GBFs are all bZIP type transcription factors which binds to DNA as a homodimer or a heterodimer.^39^ Possibility of heterodimer formation of HY5 with another bZIP proteins provides complex situations regarding signal transduction to the target site. In addition to these bZIP proteins, G-box is also known to be recognize by PIF proteins, and their binding is supposed to suppress transcription in the dark, which supports light-dependent transcriptional activation.^20^

It is not known if Element B is recognized by transcription factor other than HY5, but no reduction of the cold stress response in *hy5* and the remaining partial response to HL and UV-B in *hy5*^14^ support this possibility.

Taken together, the function of Element B, as in the case of G-box, is suggested as not just the recognition site of HY5 but rather of multiple transcription factors including HY5. Moderate sequence difference between G-box and Element B would cause moderate difference of the corresponding transcription factors between them, providing the roles of G-box and Element B toward photomorphogenesis and photoprotection, respectively. It is interesting that HY5 is still involved in both photomorphogenesis and photoprotection by binding to both G-box and Element B.

The native *ELIP2* promoter contains two HY5 binding sites, which are Element B (5’-GCCACGCC-3’) and B’ (5’-GACGTGGC-3’). This was in part shown by loss of the UV-B response by disruption of both binding sites in the context of native promoter.^14^

Considering the difference in the sequence of Element B from G-box, there might be a possibility of heterodimer formation of HY5 at Element B. One idea is heterodimer formation of HY5 with an environmental stress-specific bZIP factor which is not identified, yet. This idea fits with the observation of stress-oriented characteristics of Element B, regarding distribution of the containing promoters in the genome and functional involvement of the stress responses.

Compared with the high correlation of the Element B-containing promoters between the three stress responses, those that had the Element A-containing promoters had lower correlation.^14^ This suggests that Element A receives the stress signals with lower specificity than Element B. Identification of the corresponding transcription factors would provide further information regarding the stress signals for Element A.

A single stressor can change several physiological conditions in plant cells. HL, for example, causes photoinhibition, ROS evolution, saturation (full reduction) of electron carriers (including plastoquinone, plastocyanin, and NADPH/NADP^+^), and the development of a pH gradient across the thylakoid membrane, all of which happens at the chloroplasts. In addition, they can further affect the activation of photorespiration, which produces ROS at the peroxisomes, and alters the metabolic flow at the peroxisomes, mitochondria, and chloroplasts. All of these changes have the potential to trigger a stress signal to Element A (signals to Element A in Figure 7(a)).

The mutation analysis presented in Figure 4 and the right part of Figure 5 suggests the complexity of Element A. Because the spectrum of the three stress responses varied depending on the mutated sequence, Element A is suggested to be recognized by multiple transcription factors with different sequence and stress specificities. Therefore, Element A also provides a fine-tuned role in the multi-stress responses for photoprotection.

## Single stressor evokes parallel signals to activate a single gene

Figure 7(b) emphasizes the convergence of two parallel signals to the *ELIP2* promoter. In the models, two distinct signals are initiated by a single stressor, HL, cold, or UV-B, and the two signals then merge at the *ELIP*2 promoter for activation.

In general, the requirement of two distinct signals, which are called an “AND” operation, is a mechanism designed to limit the response only to a continuous signal, thereby avoiding the mis-activation of an intermittent signal. Therefore, one interpretation of the double input system for *ELIP2* activation is that it suppresses the transcriptional noise for the *ELIP2* expression that can be triggered by environmental fluctuation. The exact advantage of the parallel signaling to the *ELIP2* promoter unit will be understood further after detailed signaling to Element A is identified.

One interesting feature of this dual activation in the unit is that there is a greater response than when activation occurs through a single element (Figure 6). A possible molecular mechanism for this is that the corresponding transcription factors for Element A and Element B act on different aspects of transcription for activation, which is achieved by tethering different activator domains to the target promoter. The high environmental response achieved by the combination may have a positive effect on the high specificity of the response. Identification of the molecular mechanisms for the regulatory unit in the *ELIP2* promoter is expected to provide a central scheme for the prediction of stress responses based on the promoter sequences in higher plants.

There are several old reports of combination of *cis*-elements for transcriptional activation by environmental signals. Puente et al. reported synthesis of light response and green tissue-specificity in the context of the synthetic promoter by combination of two *cis*-elements including the GATA motif.^16^ In another report, ABA response of the barley *HVA1* promoter was shown to be achieved by combination of ABRE and Coupling Element (CE).^15^ Regarding ABRE, functionality of 6×ABRE without CE in transgenic Arabidopsis is recently reported.^40^ This suggests that ABRE does receive ABA signal, but it would not be enough alone to activate the *HVA1* promoter, showing similarity to Element B and A in the *ELIP2* promoter.

Currently, it is not known how common the combinational trait is in achieving signal response of plant promoters. Interestingly, prediction of stress response based on promoter sequence was more precise considering two *cis*-elements for functionality.^41^ These attempts may imply existence of many examples of the combinational units in plant genomes.

## Supplementary Material

Supplemental Material
