# Supplementary material for: Requirement of two simultaneous environmental signals for activation of Arabidopsis ELIP2 promoter in response to high light, cold, and UV-B stresses
Source: Plant Signal Behav. 2024 Aug 12;19(1):2389496. doi: 10.1080/15592324.2024.2389496 (PMC11321413; doi:10.1080/15592324.2024.2389496)
Supplement: Supplemental Material [file KPSB_A_2389496_SM6306.zip › Supplementary Figure.pdf]

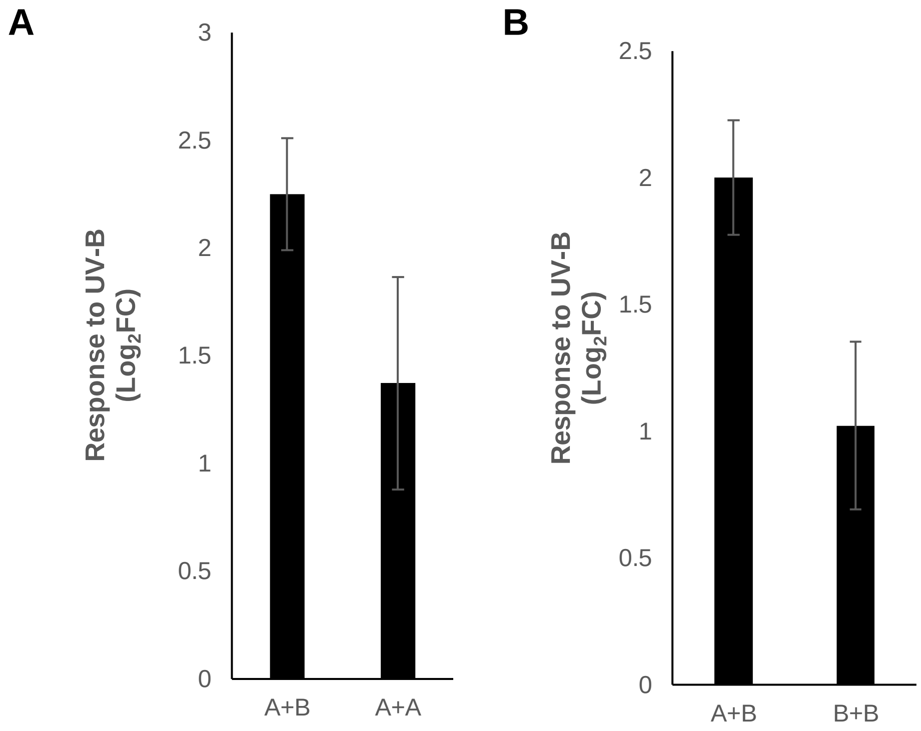

Fig S1. RT-qPCR analysis of reporter gene expression of synthetic promoters

Fold change of the UV-B response was calculated after normalization with the corresponding transcript level of an internal control (*UBQ5*). The average of Log_2(Fold Change) and standard errors are presented in the graphs. Results shown in Panels A and B are obtained from different sets of experiments.
